# A tentative list of reptilian fauna of Algeria and their conservation status

**DOI:** 10.3897/BDJ.12.e120471

**Published:** 2024-04-29

**Authors:** Rachid Rouag, Nadia Ziane, Marcos De Sousa

**Affiliations:** 1 Environmental Sciences and Agroecology Laboratory. Chadli Bendjedid University, El Tarf, Algeria Environmental Sciences and Agroecology Laboratory. Chadli Bendjedid University El Tarf Algeria; 2 Laboratory of Environmental Biosurveillance Faculty of Sciences, Department of Biology. Badji Mokhtar University, Annaba, Algeria Laboratory of Environmental Biosurveillance Faculty of Sciences, Department of Biology. Badji Mokhtar University Annaba Algeria; 3 Museu Paraense Emílio Goeldi, Belém, Brazil Museu Paraense Emílio Goeldi Belém Brazil

**Keywords:** checklist, reptiles, conservation, Algeria, Chelonia, Sauria, Ophidia

## Abstract

**Background:**

Algeria is situated at the crossroads between Europe and Africa. The northern part of the country is listed as an area of high biodiversity. Currently, the ecosystems (rivers, lakes, deserts, forests etc.) and the species are under more pressure than ever. The impact of humans is significant and many factors constitute a strong threat to this fauna, especially reptiles, which are the most vulnerable because of their low mobility. Thus, pollution, the drying up of wetlands and their conversion to agriculture have clearly affected the existence of many species. The herpetofauna of Algeria is one of the most diversified in the Mediterranean Basin, consisting of 104 species of which 16.98% are endemic. We suppose that the present list of reptilian fauna provided in this paper is not exhaustive and it is expected to include more species given the lack of research on reptiles in Algeria and its large area.

**New information:**

Our dataset contains information on reptile occurrences in Algeria. The dataset is based on original research by the staff of the Laboratory of Environmental Sciences and Agroecology of Chadli Bendjedid University in Algeria. The conservation status of all recorded species is given.

## Introduction

The documentation of herpetofauna of Algeria began a century and a half ago with the publication of the first notes by Gervais (1835), Gervais (1836) and Lataste (1880). The most important contribution in this period is from Boulenger (1891), who published a catalogue on the reptiles and amphibians of the "Barbarie", based chiefly upon the notes and collections made between 1880 and 1884 by M. Fernand Lataste. The document contains the identification keys of 62 species of reptiles listed with a precise description of every species and its distribution, but does not cover the entire geographical extent of Algeria. Later, Olivier (1894) published his document entitled "Herpétologie algérienne" or a catalogue of the reptiles and batrachians observed in Algeria. Doumergue (1901) published an essay on the herpetological fauna of Oranie with analytical tables and notes for the determination of all reptiles and batrachians of Morocco, Algeria and Tunisia. This work contains dichotomous tables that are very useful for the determination of the species. Beyond this period, the only published works, namely, the works of Gauthier (1965), Gauthier (1967), Grenot and Vernet (1972) and Grenot and Vernet (1973) were concerned mainly with Saharan herpetofauna. Currently, research on Algerian herpetological fauna is regaining interest with the appearance of many publications on the biology and ecology of some species (Benounnas 2021, Bezaz 2021, Mouane 2021, Rouag et al. 2016, Bouam et al. 2016, Mamou et al. 2016, Dellaoui et al. 2015, Rouag et al. 2007, Rouag et al. 2006, Rouag and Benyacoub 2006, Chirio and Blanc 1997, Chirio 1995, Laurent 1990). Additionally, taxonomic revision, based on molecular or morphological analyses recorded in Algeria and the Maghreb, has been the object of several publications in the last few years, in order to trace the biogeographic history of the Mediterranean herpetofauna (Beddek et al. 2018, Merabet et al. 2016, Tamar et al. 2016, Kapli et al. 2014, Stuckas et al. 2014, Anadón et al. 2012, Geniez et al. 2011, Fritz et al. 2009, Geniez et al. 2004, Geniez and Foucart 1995, Busack and McCoy 1990).

Situated in North Africa, Algeria comprises 2,381,740 km^2^ of land, more than 80% of which is desert. Algeria’s climatic regions and landscapes can be divided into four sections that run parallel to each other down the length of the country. The northernmost division, the Tell, is a coastal chain of mountains that extends from the northwest to the northeast over a distance of 1500 km. This chain contains the most important mountains (Ouarsenis, Atlas Blidéen, Djurdjura, Babors and Kroumerie). The altitudes of some summits are over two thousand metres (2308 m for Djurdjura). The diversity of relief and exposure allows the presence of a wide variety of bioclimates ranging from semi-arid in the highlands to humid in the coastal mountainous chains, always characterised by mild and humid winters and hot and dry summers (Daget 1977). The mountain range becomes High Plateaus, a massive area of mostly barren plains. The next landscape band is made up of further mountain ranges that are part of Algeria’s Saharan Atlas range, which is also the largest section. Certain sections of the Sahara Desert may not receive rain for periods of up to 20 years and the temperatures can exceed 55ºC. Rainfall in the northern areas of Algeria measures approximately 1000 mm annually.

The extensive size of Algeria, coupled with uncharted regions, challenging accessibility and a scarcity of field herpetologists, contribute to the limited availability of information on reptiles in the country. The aim of this study is to summarise all possible sources of occurrence records for reptiles in Algeria, including our data, published literature records, verified reports on social networks and records published in online databases.

## Materials and methods

The present checklist is based on the available taxonomic and faunistic literature concerning the Algerian and North African herpetofaunas (Gherbi et al. 2023, Nouira et al. 2022, Bouazza et al. 2021, Martínez‐Freiría et al. 2021, Mouane 2021, Thomson 2021, Geniez et al. 2000, Fritz and Schmidtler 2020, Beddek et al. 2018, Kindler et al. 2017, Martínez-Freiría et al. 2017, Rato et al. 2016, Metallinou et al. 2015, Geniez 2015, Wagner et al. 2011, Fonseca et al. 2009, Wilms et al. 2009, Carretero 2008, Crochet et al. 2008, Rouag and Benyacoub 2006, Carranza et al. 2004, Bons and Geniez 1996, Schleich et al. 1996, Nouira 1995, Chirio 1995, Chirio and Blanc 1993, Nilson and Andrén 1988, Nouira and Blanc 1986). We also mainly used the checklists, the reports on the herpetofauna of the Mediterranean Basin and the field guides of the Reptiles for other regions of the Western Palearctic (Rhodin 2021, Trape et al. 2012, Cox et al. 2006, Trape and Mané 2006, Bons and Geniez 1996, Nouira and Blanc 1993, Gruber 1992, Mebs 1991, Blanc 1980, Bischoff 1981, Bons 1967, Guibé 1950) We collected reptilian species occurrence records through direct field observations, especially in the north-east of Algeria. A review of scientific literature published on the herpetofauna in different regions of Algeria was used to establish the list of reptiles. We used online databases, such as Inaturalist; the Global Biodiversity Information Facility (GBIF 2023); the Reptile Database (Uetz 2021) and also the IUCN Red List (IUCN 2023). Additionally, we used personal observations of the experts, unpublished reports and consultation with specialised groups on biodiversity and wildlife in Algeria. In all, 104 species were identified.

### Geographic coverage

**Description**: Our database included all reptile species present in Algeria.

**Coordinates**: 28.033886 N; 1.659626 E.

### Quality control

The aim of this work is to provide a checklist of Algerian reptiles, based on all studies published by researchers to date. The species were identified by comparison with the bibliography and with material from collections previously identified by specialists. All assessments were made at the taxonomic level of the species. We based our analysis on the lists of Scleich et al. (1996) and Beddek (2017) and a combination with data available on databases, such as the Reptile Database (Uetz 2021), the IUCN Red List (IUCN 2023), as well as the Inaturalist platforms and Global Biodiversity Information Facility (GBIF) where species identification has been confirmed by renowned specialists. We also consulted the websites of the Muséum d'histoire naturelle de Paris (MNHN) and the Musuem of Vertebrate Zoology (California Academy of Science). Recently-cited species were only added if they appeared in publications in specialised and indexed journals. Recent studies in molecular biology allowing the description of new species were also used to update the checklist.

### Temporal coverage

**Data range**: 1835-1-01 - 2024-1-03.

### Taxonomic coverage

Reptiles constitute a major component of vertebrates in Algeria, as is the case in all hot and arid countries. This class includes 104 species belonging to four orders of reptiles: Amphisbaenia (amphisbaenians); Ophidia (snakes); Sauria (lizards); and Chelonia (turtles and tortoises). However, the majority of species are lizards (67.62%) and snakes (25.71%) (Table 1). The most important reptile families in the region are Lacertidae (24 species), Gekkonidae (Geckos: 18 species), Scincidae (Skinks: 15 species) and Colubridae (Colubridae: 12 species).

The desert crocodile species *Crocodylussuchus* (Geoffroy Saint-Hilaire, 1807), which once inhabited the Algerian Sahara, is now considered extinct. This species ceased to exist in the Immidir and Ahaggar Regions of Algeria since the early 20^th^ century, as noted by Lescure (2014).

Table 1

## Checklists

### Checklist of Reptiles of Algeria

#### Mauremys leprosa

(Schweigger, 1812)

1FEFE5E4-7C4F-53D2-A407-59CDF7601083

##### Conservation status

VU

##### Distribution

The north of the country up to the limit of the Saharan Atlas (El Kala, Oum El Bouaghi, Alger, Oran, Laghouat).

##### Notes

Bezaz (2021); Bakhouche et al. (2019); Rouag and Benyacoub (2006); Schleich et al. (1996); Doumergue (1901) .

#### Emys orbicularis

(Linnaeus, 1758)

73A77719-67E6-5B53-89E3-33A87EBDE3F9

##### Conservation status

NT

##### Distribution

Coastal strip in the east of the country (El Kala, Annaba, Jijel, Skikda, Béjaia).

##### Notes

Gherbi et al. (2023); Rouag and Benyacoub (2006); Samraoui and de Belair (1997)

#### Testudo graeca

Linnaeus, 1758

119D5958-46CA-5D2F-9E55-2EF8DB5B1DD0

##### Conservation status

VU

##### Distribution

The north of the country up to the limit of the Saharan Atlas.

##### Notes

Gherbi et al. (2023); Bezaz (2021); Rouag and Benyacoub (2006); ; Schleich et al. (1996)

#### Caretta caretta

(Linnaeus, 1758)

D013E0DB-400E-5D47-985B-F44E7BA1015B

##### Conservation status

VU

##### Distribution

Along the Algerian coast (Algiers, Skikda, Jijel, El Tarf, Mostghanem, Oran).

##### Notes

Bennounas and Bennounas (2020); Belmahi et al. (2020); Laurent (1990); Lallemant (1867).

#### Chelonia mydas

(Linnaeus, 1758)

3390A32C-1700-5A8F-A8FF-89220401C3BF

##### Conservation status

EN

##### Distribution

Rarest species (Ain Temouchent).

##### Notes

Bennounas and Bennounas (2020); Belmahi et al. (2020).

#### Dermochelys coriacea

(Vandelli, 1761)

3B24C9D2-1187-5168-8490-09FBCC3BC834

##### Conservation status

VU

##### Distribution

Along the Algerian coast (Algiers, Skikda, Béjaia, Jijel, Boumerdes, Oran, Ain Temouchent).

##### Notes

Bennounas and Bennounas (2020); Belmahi et al. (2020).

#### Tarentola mauritanica

(Linnaeus, 1758)

6402522E-6FEC-56F4-8987-1051C9B06829

##### Conservation status

LC

##### Distribution

The north of the country up to the limit of the Saharan Atlas (Oran, Aîn sefra, Mechria, El Kala, Annaba).

##### Notes

Benelkadi et al. (2021); Rouag and Benyacoub (2006); Doumergue (1901).

#### Tarentola deserti

Boulenger, 1891

195B78E3-1F35-5B0E-A8DE-2CC50A511CEC

##### Conservation status

LC

##### Distribution

the Saharan Atlas and the High Plateaus.

##### Notes

Joger et al. (2006); Schleich et al. (1996).

#### Tarentola annularis

(Geoffroy Saint-Hilaire, 1827)

E86E0B32-5C43-51C2-A7FD-755881330866

##### Conservation status

LC

##### Distribution

Relictual species in the Sahara near Tindouf.

##### Notes

Gauthier (1967) .

#### Tarentola hoggarensis

Werner, 1937

C27E59A6-64B0-52E5-A07E-F1217A1DC20A

##### Conservation status

LC

##### Distribution

Ahaggar and Tassili n’Ajjer. Isolated populations exist near Tindouf.

##### Notes

Trape et al. (2012); Schleich et al. (1996)

#### Tarentola neglecta

Strauch, 1887

AB64C43D-F1A9-52E1-9375-E0C54309244F

##### Conservation status

LC

##### Distribution

Aurès, Biskra, Touggourt, Ouargla.

##### Notes

Trape et al. (2012); Doumergue (1901); Boulenger (1891).

#### Ptyodactylus ragazzii

Anderson, 1898

D017BDCF-B3E5-52AC-904B-B625A33FA405

##### Conservation status

LC

##### Distribution

The south (Ahaggar, Tassili).

##### Notes

Metallinou et al. (2015); Trape et al. (2012); Schleich et al. (1996).

#### Ptyodactylus oudrii

Lataste, 1880

212752DC-583E-5748-92EF-8E04002512DE

##### Conservation status

LC

##### Distribution

Aurès, Bou saada, Beni Ouenif, Ghardaïa, Laghouat, El Goléa, Biskra, Béni Abbès, Aïn Sefra.

##### Notes

Le Berre (1989).

#### Hemidactylus turcicus

(Linnaeus, 1758)

D0BB1AE1-301A-5090-84D1-DDD4146A4D6F

##### Conservation status

LC

##### Distribution

El Kala, Oran, Algiers, Annaba, Oum El Bouaghi.

##### Notes

Bezaz (2021); Rouag and Benyacoub (2006); Schleich et al. (1996); Boulenger (1891).

#### Stenodactylus sthenodactylus

(Lichtenstein, 1823)

6888485B-72B0-595B-83E3-9FD60F5AB089

##### Conservation status

DD

##### Distribution

Aguelmane Assar (Tassili n'Ajjer), M'sila.

##### Notes

Benelkadi et al. (2021); Metallinou et al. (2012)

#### Stenodactylus mauritanicus

Guichenot, 1850

E5F86AC4-8EC8-59EF-8479-00928770E035

##### Conservation status

LC

##### Distribution

Oran; Tindouf, Aïn Séfra, Biskra, Ghardaïa, Bou Saada, Ouargla.

##### Notes

Schleich et al. (1996); Doumergue (1901); Boulenger (1891); Guichenot (1850).

#### Stenodactylus petrii

Anderson, 1896

4A5B5E43-5A7A-5FE7-990A-58BD86B184FF

##### Conservation status

LC

##### Distribution

Tindouf, Touggourt, M'raier, Aïn Séfra, Biskra, Ghardaïa, Zelfana, Bou Saada, El Goléa, Ouargla.

##### Notes

Schleich et al. (1996).

#### Tropiocolotes tripolitanus

Peters, 1880

0D288233-AA37-5767-969F-574B09B4B78F

##### Conservation status

LC

##### Distribution

Tindouf, Ahaggar, Biskra, Figuig and Kenadsa.

##### Notes

Schleich et al. (1996).

#### Tropiocolotes algericus

Loveridge, 1947

634450E9-C4B1-56E7-9CF4-491905CA35A5

##### Conservation status

LC

##### Distribution

Ahaggar, Tindouf, Biskra, Kenadsa.

##### Notes

Trape et al. (2012) .

#### Tropiocolotes nubicus

Baha El Din, 1999

0E82F39A-8C7B-5F97-A087-97C16DFDFF4A

##### Conservation status

LC

##### Distribution

Southern Algeria (Tassili n’Ajjer and Ahaggar).

##### Notes

Machado et al. (2021) .

#### Tropiocolotes chirioi

Ribeiro-Júnior, Koch, Flecks, Calv & Meiri, 2022

24FB8D81-91A8-5A79-82C1-6E1F34F40FA1

##### Conservation status

DD

##### Distribution

North-eastern Algeria (the Aurès Mountains).

##### Notes

Ribeiro-Júnior et al. (2022) .

#### Tropiocolotes tassiliensis

Ribeiro-Júnior, Koch, Flecks, Calv & Meiri, 2022

D4EA9EB4-941D-57F1-8567-1BF1D7B879B8

##### Conservation status

DD

##### Distribution

Southern Algeria (Tassili n’Ajjer and Ahaggar).

##### Notes

Ribeiro-Júnior et al. (2022) .

#### Cyrtopodion scabrum

(Heyden, 1827)

C679E345-D50B-5F38-BAAB-6C2013D6BC14

##### Conservation status

DD

##### Distribution

North-eastern Sahara (El Oued). South-eastern Algeria (El Menea and Ouargla Province)

##### Notes

Mouane (2022); Sadine et al. (2021); Mouane (2020).

#### Saurodactylus mauritanicus

(Duméril and Bibron, 1836)

028D6B1E-4EC0-5960-801C-2B53F9778170

##### Conservation status

LC

##### Distribution

Djebel Mizab (sebdou), Tiaret, Alger. A relict population north of Ghardaïa.

##### Notes

Le Berre (1989) .

#### Chalcides minutus

Caputo, 1993

BEA771F6-B6A9-5591-AF57-D37A52FC0D11

##### Conservation status

VU

##### Distribution

Théniet El Had National Park.

##### Notes

Montero-Mendieta et al. (2017) .

#### Chalcides ocellatus

(Forskål, 1775)

763C1228-4DDD-5977-B263-C791FFEC6CFD

##### Conservation status

LC

##### Distribution

Oum El Bouaghi, Oran, El Oued, El Kala, Constantine.

##### Notes

Bezaz (2021); Rouag and Benyacoub (2006); Schleich et al. (1996); Gauthier (1967).

#### Chalcides parallelus

Doumergue, 1901

C997D9B5-F6E0-5F40-B6CC-2C81B607DD5A

##### Conservation status

EN

##### Distribution

North-western Algeria (occurring mainly along a narrow coastal strip).

##### Notes

Beddek (2017) .

#### Chalcides mauritanicus

(Lataste and Rochebrune, 1876)

1342BF62-83CC-538F-8FF7-91042396A171

##### Conservation status

EN

##### Distribution

Coastal districts of north-western Provinces (Oran).

##### Notes

Sindaco et al. (2013); Schleich et al. (1996); Doumergue (1901); Boulenger (1891).

#### Chalcides chalcides

(Linnaeus, 1758)

2D241888-6DBC-516C-B035-71BB18B7FFD1

##### Conservation status

DD

##### Distribution

Only one observation in the El Kala National Park.

##### Notes

Rouag and Benyacoub (2006) .

#### Chalcides mertensi

Klausewitz, 1954

B78DB7B0-9D6F-59F7-BB4A-C9AACD966C54

##### Conservation status

LC

##### Distribution

Forest areas in northern Algeria.

##### Notes

Benelkadi et al. (2021); Rouag and Benyacoub (2006); Caputo et al. (1993).

#### Chalcides boulengeri

Anderson, 1892

26C25E86-A597-500A-8BE0-AA38835856D1

##### Conservation status

LC

##### Distribution

Southern Algeria (Tassili n'Ajjer and Ahaggar).

##### Notes

Uetz (2021); Trape et al. (2012).

#### Chalcides delislei

(Lataste and Rochebrune, 1876)

349B84C2-E3F6-598D-BCE5-F965F09CA21B

##### Conservation status

LC

##### Distribution

Southern Algeria (Tassilli n'Ajjer et Ahaggar).

##### Notes

Uetz (2021); Beddek (2017).

#### Heremites vittatus

(Olivier, 1804)

F0C694B5-B735-5230-B374-C67E5A7F8344

##### Conservation status

LC

##### Distribution

Oued Souf; Biskra.

##### Notes

Boulenger (1891); Lallemant (1867).

#### Scincus scincus

(Linnaeus, 1758)

ADBFF0E8-54CD-5BFE-9EBB-8289704F546C

##### Conservation status

LC

##### Distribution

Oued Souf, Touggourt, Ouargla.

##### Notes

Lallemant (1867) .

#### Eumeces algeriensis

Peters, 1864

006E4AC7-99F8-5DFC-AE45-D51965EDE517

##### Conservation status

LC

##### Distribution

The northwest (Oran; Aïn-Temouchent).

##### Notes

Schleich et al. (1996); Doumergue (1901); Strauch (1862).

#### Eumeces schneideri

(Daudin, 1802)

DBD0B181-F1AC-5446-B988-5D0B98F74F93

##### Conservation status

LC

##### Distribution

North-eastern Algéria.

##### Notes

Schleich et al. (1996) .

#### Scincopus fasciatus

(Peters, 1864)

6B813D01-42C3-52D3-A7D9-F5A8434B1768

##### Conservation status

LC

##### Distribution

Relictual species in Touggourt and Biskra.

##### Notes

Doumergue (1901); Schleich et al. (1996).

#### Scincus albifasciatus

Boulenger, 1890

FEFC447D-62EF-5C21-8C66-4015C99E122D

##### Conservation status

LC

##### Distribution

Tassili n'Ajjer, d’El-Meniaa (El-Goléa).

##### Notes

Schleich et al. (1996).

#### Trachylepis quinquetaeniata

(Lichtenstein, 1823)

63D1779E-AB6D-5449-8E7E-5E47AB5B14A2

##### Conservation status

NE

##### Distribution

Only one observation in El Hamdania (50 km au sud d’Alger).

##### Notes

Rouag (2016).

#### Acanthodactylus erythrurus

(Schinz, 1833)

F1F59B5C-513C-5208-A525-F2B137B57494

##### Conservation status

LC

##### Distribution

Northern Algeria (El Kala, Oran, Algiers, Bordj-Bou-Arrerij, Tebessa, M'sila).

##### Notes

Benelkadi et al. (2021); Rouag (2016); Boulenger (1891).

#### Acanthodactylus boskianus

(Daudin, 1802)

E95308F9-0BEF-55DB-86DE-01E490C9D2E8

##### Conservation status

LC

##### Distribution

Arid and Saharan areas (Ghardaia, Berrian, Laghouat, Bou-Saada).

##### Notes

Tamar et al. (2016); Sindaco et al. (2013); Trape et al. (2012); Schleich et al. (1996); Boulenger (1891).

#### Acanthodactylus maculatus

(Gray, 1838)

FF4ECF2F-ED16-5880-9E38-C7FFF0B3D674

##### Conservation status

LC

##### Distribution

Covers the north of the country in the High Plateaus.

##### Notes

Tamar et al. (2016); Schleich et al. (1996); Salvador (1982);Doumergue (1901).

#### Acanthodactylus scutellatus

(Audouin, 1827)

69E21E62-41CD-53AD-A2A5-6F59C2090E04

##### Conservation status

LC

##### Distribution

Béni Abbès, Béchar, In Salah, Tassili, Touggourt, Ouargla, Biskra, Laghouat, Bou Saada.

##### Notes

Schleich et al. (1996); Salvador (1982); Boulenger (1891).

#### Acanthodactylus dumerilii

(Miline-Edwards, 1829)

27DF4F97-E6F0-5762-B169-CFDDCDA821F5

##### Conservation status

LC

##### Distribution

Ergs of the north of the Sahara (Biskra, Tougourt, Laghouat and Bou Saada).

##### Notes

Trape et al. (2012); Wilms et al. (2009); Crochet et al. (2008); Schleich et al. (1996); Boulenger (1891).

#### Acanthodactylus longipes

Boulenger, 1918

002A5889-9098-5856-9007-612C98FC970B

##### Conservation status

LC

##### Distribution

Ergs of the Sahara (Oued Souf, Ouargla).

##### Notes

Schleich et al. (1996); Salvador (1982).

#### Acanthodactylus bedriagai

Lataste, 1881

19A6B8BF-4B09-5A62-9417-5BDCF2E66B3D

##### Conservation status

NT

##### Distribution

The Oriental Plateaux of Algeria (Constantine, Setif; Batna, Oum El Bouaghi).

##### Notes

Bezaz (2021); Salvador (1982); Doumergue (1901).

#### Acanthodactylus savignyi

(Audouin, 1827)

32ECC374-D3AE-58D1-8AF6-AA0E4A0D21DB

##### Conservation status

NT

##### Distribution

Dune beaches of Mostaganem, Oran and Ain T’émouchent.

##### Notes

Beddek (2017); Doumergue (1901).

#### Acanthodactylus spinicauda

Doumergue, 1901

69AE334B-4293-5C60-9913-6A3A6D0D4007

##### Conservation status

CR

##### Distribution

El Abiod Sidi Cheikh.

##### Notes

Dellaoui et al. (2015); Doumergue (1901).

#### Acanthodactylus taghitensis

Geniez and Foucart, 1995

47B6196F-3750-58AA-BD9C-26BA25C5CFF1

##### Conservation status

DD

##### Distribution

Tindouf and Taghit.

##### Notes

Geniez and Foucart (1995) .

#### Acanthodactylus blanci

Doumergue, 1901

8B8A0680-8FC1-5883-B2A7-BC77B0F2F34D

##### Conservation status

EN

##### Distribution

Eastern Algeria extending from Tebessa to Algiers.

##### Notes

Miralles et al. (2020); Boulenger (1891).

#### Psammodromus algirus

(Linnaeus, 1758)

65530E9A-2B4F-57FC-B82B-E6AFBEB95D39

##### Conservation status

LC

##### Distribution

Northern Algeria (Annaba, El Kala, Oum El Bouaghi, M'sila, Oran).

##### Notes

Benelkadi et al. (2021); Rouag and Benyacoub (2006); Schleich et al. (1996); Boulenger (1891) .

#### Psammodromus blanci

(Lataste, 1880)

7E36D398-8CD7-5B7B-969E-F3A72E78B930

##### Conservation status

NT

##### Distribution

The Aurès and the steppes of the High Plateaus.

##### Notes

Beddek et al. (2018); Trape et al. (2012).

#### Ophisops occidentalis

(Boulenger, 1887)

9FB73CF2-CC27-51ED-815B-A54E2A492B9F

##### Conservation status

LC

##### Distribution

The Saharan Atlas (Batna, Oum El Bouaghi, Tebessa).

##### Notes

Bezaz (2021); Schleich et al. (1996); Boulenger (1891).

#### Ophisops elegans

Menetries, 1832

A9CBF174-352C-5DF6-AD63-CEC02FF63BFD

##### Conservation status

DD

##### Distribution

The Saharan Atlas (Aurès).

##### Notes

Schleich et al. (1996); Chirio and Blanc (1993).

#### Mesalina olivieri

(Audouin, 1829)

8186D440-231A-5CC9-9FF9-B1E30DAED3D0

##### Conservation status

LC

##### Distribution

Steppes and arid regions (Oran, Oum El Bouaghi).

##### Notes

Bezaz (2021); Pizzigalli et al. (2021); Trape et al. (2012); Schleich et al. (1996); Doumergue (1901); Boulenger (1891).

#### Mesalina guttulata

(Lichtenstein, 1823)

CE5191F9-8781-531E-8FED-303435AC2E61

##### Conservation status

LC

##### Distribution

Tlemcen, Bou Saada; Mecheria, Saïda, Béni Abbès, Erg Occidental, Erg Chech, Laghouat, Ahaggar.

##### Notes

Trape et al. (2012); Schleich et al. (1996); Doumergue (1901); Strauch (1862).

#### Mesalina pasteuri

(Bons, 1960)

5403BA42-ADFF-5281-984B-649BB57F0824

##### Conservation status

LC

##### Distribution

The dunes of ergs (Beni Abbès, Amguid, Ahaggar).

##### Notes

Trape et al. (2012); Gauthier (1967); Gauthier (1965) .

#### Mesalina rubropunctata

(Lichtenstein, 1823)

4B631306-60F8-510F-A42F-469B1B7FAFCE

##### Conservation status

LC

##### Distribution

In the Sahara, in arid areas with stony or rocky soil, rarely sandy.

##### Notes

Trape et al. (2012); Gauthier (1967); Gauthier (1965); Seurat (1930).

#### Timon pater

(Lataste, 1880)

BF37D1D4-65E5-5610-A833-04661305B9E4

##### Conservation status

LC

##### Distribution

North of Algeria (Khroumirie, High Plateaus, Aures, Kabylie).

##### Notes

Uetz (2021); Schleich et al. (1996).

#### Timon tangitanus

(Boulenger, 1889)

F66DFCF2-B043-5F9B-A46D-55DDC8EF5D1A

##### Conservation status

LC

##### Distribution

North-west of Algeria (Senalba in Djelfa, Stiten, El Bayad).

##### Notes

Beddek (2017).

#### Podarcis vaucheri

(Boulenger, 1905)

CF04CB39-787C-5549-BE13-CA63F5EAF642

##### Conservation status

LC

##### Distribution

Tlemcen, Constantine, Setif, Beni Mansour, Tebessa, Oran, El Kala, Oum El Bouaghi.

##### Notes

Bezaz (2021); Rouag and Benyacoub (2006) Strauch (1862); Boulenger (1891); Doumergue (1901).

#### Scelarcis perspicillata

(Duméril and Bibron, 1839)

06A68DF1-CFFC-5C6A-A3FF-8019CB6A720C

##### Conservation status

LC

##### Distribution

Northwest of Algeria (Oran).

##### Notes

Uetz (2021); Doumergue (1901).

#### Philochortus zolii

Scortecci, 1934

C5890493-703C-5ECA-9576-1AB55918B144

##### Conservation status

DD

##### Distribution

Tamanrasset (on the road to Adriane and in the Municipality of Tagmart-East

##### Notes

Scheinberg and Fong (2024); Haddad et al. (2024).

#### Chamaeleo chamaeleon

(Linnaeus, 1758)

FE8C9981-90F4-5716-8F5F-36146E4C1E3E

##### Conservation status

LC

##### Distribution

The north (Oum El Bouaghi, M'sila, Oran).

##### Notes

Bezaz (2021); Benelkadi et al. (2021); Trape et al. (2012); Schleich et al. 1996; Boulenger (1891); Doumergue (1901).

#### Varanus griseus

(Daudin, 1803)

1B02A84A-E741-5429-92B7-7AA90BDF38ED

##### Conservation status

LC

##### Distribution

Occupies the entire Sahara.

##### Notes

Benelkadi et al. (2021); Trape et al. (2012); Schleich et al. (1996); Boulenger (1891); Doumergue (1901).

#### Hyalosaurus koellikeri

(Günther, 1873)

B2AD4282-F1C1-5202-B8CB-F77DA3D36E4E

##### Conservation status

DD

##### Distribution

Northwest of Algeria (Tlemcen Mountains).

##### Notes

Mateo et al. (1998).

#### Uromastyx acanthinura

Bell, 1825

74B8E143-E93E-5A14-97AB-5A28D991CCB9

##### Conservation status

LC

##### Distribution

Central and eastern Algeria (Tlemcen, Bou-Saada and the Mzab; Mecheria, Saïda).

##### Notes

Trape et al. (2012); Doumergue (1901); Strauch (1862).

#### Uromastyx alfredschmidti

Wilms and Böhme, 2001

B4A9B063-7050-558F-9701-1243B8AE1EDE

##### Conservation status

NT

##### Distribution

South-eastern Algeria and bordering regions of Libya (Tassili n' Ajjer).

##### Notes

Tamar et al. (2017); Trape et al. (2012).

#### Uromastyx dispar

Heyden, 1827

CB91F48A-28C5-578E-B650-C117740CACFA

##### Conservation status

LC

##### Distribution

*Uromastyxdisparmaliensis* Joger and Lambert, 1996 in south-western Algeria (Taoudrart en Tanezrouft). *Uromastyxdisparflavifasciata* Mertens, 1962 in Tindouf.

##### Notes

Trape et al. (2012); Wilms et al. (2009); Wilms et al. (2009).

#### Uromastyx geyri

Müller, 1922

20AA1AE6-CDBE-5523-91F9-C73FC31F5E5A

##### Conservation status

NT

##### Distribution

Southern Algeria (Ahaggar and Tassili n’Ajjer).

##### Notes

Trape et al. (2012) .

#### Uromastyx nigriventris

Rothschild and Hartert, 1912

F52A07EB-E71D-5664-8D86-4433E39124A0

##### Conservation status

LC

##### Distribution

Western Algeria (du Mzab au Guir).

##### Notes

Trape et al. (2012).

#### Trapelus tournevillei

(Lataste, 1880)

9FCCA73D-C39F-527E-915A-FA44B72D4EC1

##### Conservation status

LC

##### Distribution

Central Algeria in ‘Erg Oriental’ (Touggourt, Ouargla), ‘Erg Occidental’ (El Goléa, Béni Abès) and ‘Erg er Raoui’.

##### Notes

Schleich et al. (1996).

#### Trapelus schmitzi

Wagner et Böhme, 2007

59E14B8C-0495-5D9E-A913-65FAEEE9C793

##### Conservation status

LC

##### Distribution

Southern Algeria (Tassili n'Ajjer).

##### Notes

Uetz (2021); Wagner et al. (2008).

#### Trapelus boehmei

Wagner et al., 2011

3E66D99F-4AC3-5380-AC95-A15B51122388

##### Conservation status

LC

##### Distribution

Sahara (Bechar).

##### Notes

Uetz (2021); Wagner et al. (2011).

#### Agama bibronii

A. Duméril in Duméril & Duméril, 1851

F5B8B200-B4A6-5266-AFE7-E0D7EEBD0B28

##### Conservation status

LC

##### Distribution

Nortwestern Algeria (Tlemcen, Bou Saada and the Mzab; Mecheria, Saïda)

##### Notes

Uetz (2021); Trape et al. (2012); Doumergue (1901); Strauch (1862).

#### Agama tassiliensis

Geniez, Padial and Crochet, 2011

040A7AF1-304E-5AA5-A494-D336FD128748

##### Conservation status

LC

##### Distribution

Tassili n'Ajjer and Ahaggar.

##### Notes

Trape et al. (2012); Geniez et al. (2011).

#### Trogonophis wiegmanni

Kaup, 1830

48D0C8A4-3806-55E7-9973-6A01B4A3DC10

##### Conservation status

LC

##### Distribution

Mostaganem, Algiers, Oran, Biskra, Oum El Bouaghi.

##### Notes

Bezaz (2021); Schleich et al. (1996); Boulenger (1891); Lallemant (1867); Strauch (1862).

#### Malpolon monspessulanus

(Hermann, 1804)

981C0756-411D-551A-B902-757D22FD171A

##### Conservation status

LC

##### Distribution

North-western Algeria (Mecheria, Saida, M'sila).

##### Notes

Benelkadi et al. (2021); Schleich et al. (1996): Doumergue (1901).

#### Malpolon insignitus

(Geoffroy Saint-Hilaire, 1827)

98D8B2D7-59A2-57F3-AA17-148B3D414C23

##### Conservation status

LC

##### Distribution

The north-eastern and the centreof Algeria (Oum El Buaghi, El Kala).

##### Notes

Bezaz (2021); Rouag and Benyacoub (2006); Schleich et al. (1996); Doumergue (1901).

#### Malpolon moilensis

(Reuss, 1834)

55A28E9A-6532-5B3D-A427-8DF78875EDAE

##### Conservation status

LC

##### Distribution

Wide distribution throughout the Algerian Sahara (Bou Saada, Biskra, El-Abiod-Sidi-Cheikh).

##### Notes

Schleich et al. (1996); Doumergue (1901); Olivier (1894).

#### Natrix astreptophora

(Seoane, 1884)

FD776E8D-E016-5F7A-8E38-3942BB38540A

##### Conservation status

LC

##### Distribution

La Chiffa, El Kala.

##### Notes

Rouag and Benyacoub (2006); Schleich et al. (1996); Doumergue (1901).

#### Natrix maura

(Linnaeus, 1758)

1C6E218A-FBC8-5F4E-9139-AE2C6B5264F1

##### Conservation status

LC

##### Distribution

Algiers, El Kala, Oum El Bouaghi.

##### Notes

Bezaz (2021); Rouag and Benyacoub (2006); Schleich et al. (1996); Lallemant (1867).

#### Hemorrhois hippocrepis

(Linnaeus, 1758)

722BEC1A-EDEE-535C-991F-3E21F34F6FAF

##### Conservation status

LC

##### Distribution

Northern Algeria (El Kala, Sebdou, Oum El Bouaghi, M'sila).

##### Notes

Benelkadi et al. (2021); Bezaz (2021); Rouag and Benyacoub (2006); Schleich et al. (1996); Doumergue (1901).

#### Hemorrhois algirus

(Jan, 1863)

D09F3210-0639-50A0-B526-E3480D325A45

##### Conservation status

LC

##### Distribution

In the North, the steppe environments (Oran, Nâama, Ain Ain Sefra, Oum El Bouaghi). In the South in Tassili.

##### Notes

Bezaz (2021); Trape et al. (2012); Doumergue (1901); Boulenger (1891).

#### Lytorhynchus diadema

(Duméril, Bibron and Duméril, 1854)

B3B42D2D-8F04-5F28-A40F-95237C4494E7

##### Conservation status

LC

##### Distribution

Bénis Abbès, El Oued, Mraïer, Sud Oranais, Souf, Ahaggar, Tassili n'Ajjer.

##### Notes

Trape and Mané (2006); Schleich et al. (1996); Boulenger (1891).

#### Spalerosophis diadema

(Schlegel, 1837)

00696AB2-C103-513F-AA44-6E6419A1D544

##### Conservation status

LC

##### Distribution

Ouargla, Biskra.

##### Notes

Doumergue (1901) .

#### Spalerosophis dolichospilus

(Werner, 1923)

05103A04-D54A-535E-A5E2-6CC3947F2DEF

##### Conservation status

DD

##### Distribution

M'sila, Beni Abbès, Ouargla, Ghardaïa, Ahaggar, Aïn Sefra, Biskra, Oued Rhir.

##### Notes

Benelkadi et al. (2021); Schleich et al. (1996); Gauthier (1967); Doumergue (1901); Boulenger 1891.

#### Telescopus obtusus

(Reuss, 1834)

66350B26-118E-577D-9616-8AAE467F2F69

##### Conservation status

DD

##### Distribution

Mertoutek (Ahaggar) and In-Sebuk Oua Mellen (Immidir).

##### Notes

Crochet et al. (2008) .

#### Telescopus tripolitanus

(Werner, 1909)

92BA8E5E-5F59-5D9F-B654-268CAE6C2CEF

##### Conservation status

DD

##### Distribution

Tindouf.

##### Notes

Crochet et al. (2008) .

#### Coronella girondica

(Daudin, 1803)

DEC0DF16-1FA7-5D5E-AD69-C1B985497D96

##### Conservation status

LC

##### Distribution

Northern Algeria (Djelfa).

##### Notes

Schleich et al. (1996); Lallemant (1867).

#### Macroprotodon abubakeri

Wade, 2001

20E5DE8A-466D-53D2-8899-24347AF7626C

##### Conservation status

DD

##### Distribution

North-western Algeria (Oran, Habibas Islands).

##### Notes

Carranza et al. (2004); Wade (2001).

#### Macroprotodon mauritanicus

Guichenot, 1850

73FA8F8C-E6CA-5981-B1E1-A7414CEA4D0E

##### Conservation status

LC

##### Distribution

North-eastern Algeria (Oum El Bouaghi).

##### Notes

Bezaz (2021); Busack and McCoy (1990).

#### Macroprotodon brevis

(Günther, 1862)

025A8682-AC53-5D9A-8BA5-F7EB705CFD28

##### Conservation status

LC

##### Distribution

Northern Sahara from the Moroccan border to the Tunisian border. Isolated population also exists in the Ahaggar in southern Algeria.

##### Notes

Geniez (2015); Carranza et al. (2004); Strauch (1862).

#### Platyceps saharicus

Schätti and McCarthy, 2004

E820D00F-DC21-5E7E-8828-03BFA9D963DE

##### Conservation status

LC

##### Distribution

Southern Algeria (Immdir Massif).

##### Notes

Geniez (2015); Geniez and Guathier (2008).

#### Psammophis schokari

(Forskal, 1775)

7F030D15-1C69-5145-ABAA-9216EF407461

##### Conservation status

LC

##### Distribution

Aïn-Sefra; Béni Ounif, Reggane, Beni-Abbès, Oum El Bouaghi, Ahggar, Djbel Aissa.

##### Notes

Benelkadi et al. (2021); Gauthier (1967); Lallemant (1867); Gervais (1836).

#### Psammophis aegyptius

Marx, 1958

A7D88E04-61C2-5BA6-845F-34E16FC98CAA

##### Conservation status

DD

##### Distribution

Tassili, Ahaggar.

##### Notes

Boulaouad (2023); Trape and Mané (2006); Schleich et al. (1996).

#### Myriopholis algeriensis

(Jacquet, 1896)

AC84BBBC-1B35-5718-B927-4A0CFCAC2020

##### Conservation status

LC

##### Distribution

Beni Abbès, Biskra, Tassili n'Ajjer.

##### Notes

Trape (2002); Baha El Din (2001); Schleich et al. (1996).

#### Naja haje

(Linnaeus, 1758)

60638E5E-BA1E-5296-BE08-FA70E644BCE5

##### Conservation status

LC

##### Distribution

Biskra, Beni Ounif, Chott Melghir, Beni Abbès, Bir El Ater.

##### Notes

Boulenger (1891) .

#### Cerastes cerastes

(Linnaeus, 1758)

28B4AA28-9833-53C1-8506-3861F4197155

##### Conservation status

LC

##### Distribution

Wide distribution throughout the Algerian Sahara (Ahaggar, Tassili n'Ajjer, Beni Ounif, Biskra, Saïda; M'sila).

##### Notes

Benelkadi et al. (2021); Schleich et al. (1996); Boulenger (1891); Strauch (1862).

#### Cerastes vipera

(Linnaeus, 1758)

3208BDD5-3D12-5BBE-9AC6-6E4B25D0A35A

##### Conservation status

LC

##### Distribution

Wide distribution throughout the Algerian Sahara (Béni Abbès Ahaggar, Tassili n'Ajjer).

##### Notes

Schleich et al. (1996); Gauthier (1967); Doumergue (1901).

#### Daboia mauritanica

Gray, 1849

989CA850-4941-53FF-A323-E33C43ED53E5

##### Conservation status

LC

##### Distribution

Between the Tellian Atlas and the Saharan Atlas (Oum El Bouaghi, Nâama, M'sila, Oran).

##### Notes

Bezaz (2021); Benelkadi et al. (2021); Schleich et al. (1996); Doumergue (1901).

#### Echis pyramidum

(Geoffroy Saint-Hilaire, 1827)

20D432F7-FFF4-5493-AB52-E5ADFB54A7BC

##### Conservation status

LC

##### Distribution

Biskra, Constantine, isolated populations in the Ahaggar and Tassili n’ajjer.

##### Notes

Geniez 2015; Trape and Mané 2006; Schleich et al. 1996.

#### Vipera monticola

Saint Girons, 1953

E74414A5-29EE-5A4D-89AA-E989302F6E45

##### Conservation status

VU

##### Distribution

Restricted to the Tell Atlas in the mountain ranges of the north of Algeria (Annaba, Jijel, Tizi Ouzou, Bejaia, Tlemcen, Skikda).

##### Notes

Martínez‐Freiría et al. (2021); Bouam et al. (2018); Schleich et al. (1996); Olivier (1894).

#### Eryx jaculus

(Linnaeus, 1758)

5F6C2071-5A39-592A-A879-3F5FB18AF63D

##### Conservation status

LC

##### Distribution

The north especially the High Plateaus and semi-arid zones (Oran, Oued Magra, Biskra, Batna, M'sila).

##### Notes

Benelkadi et al. (2021); Schleich et al. (1996); Boulenger (1891); Strauch (1862).

## Analysis

### Conservation status of Reptiles

In this study, all the reptile species were evaluated for their global conservation status according to the IUCN system. This status represents an important tool with regard to identifying priorities for species conservation. All reptile species in Algeria are included in the IUCN Red List. As very few works cover the distribution of reptiles in Algeria, this evaluation is only approximate and requires a continuous update of the data. The number of species in the different IUCN Red List Categories is shown in Table 2.

**Figure 1.**
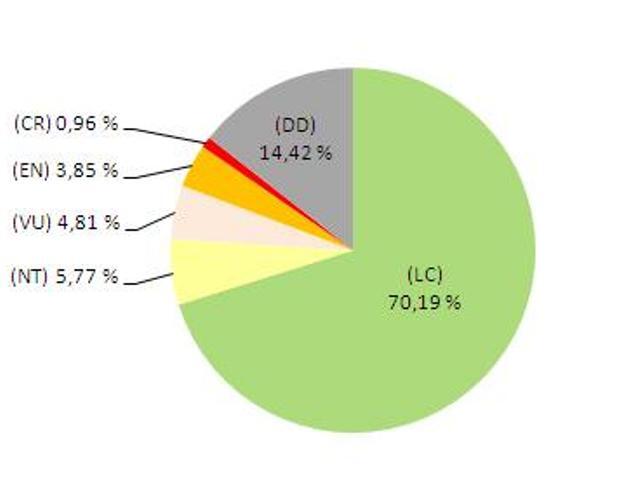
Summary of the conservation status of reptiles in Algeria.

To summarise, 30.19% of Algerian reptile species are globally threatened, with 0.96% Critically Endangered, 3.85% Endangered, 4.81% Vulnerable and 5.77% Near Threatened. A total of 70.19% (73 species) were assessed as Least Concern and 15 (14.42%) species were considered to be Data Deficient. It should be mentioned that all species of Testudines are threatened (Fig. 1).

### Endemic status of Reptiles

The richness of the Algerian herpetofauna is a result of various factors, including geographical, climatic and topographical conditions. These factors have further contributed to the isolation and diversification of many taxa which has allowed the presence of several species that are endemic to Algeria or that are shared with Tunisia, Morocco or Libya. A total of 16.98% of the Reptiles in Algeria (18 species) are endemic species to the Maghreb, of which 22.22% are endemic to Algeria and are represented by four species of lizards (Table 3).

### Hotspots for herpetological diversity

To highlight the most important areas for reptile diversity, information related to the status of the different species was compiled. Thus, the different criteria, namely endemism, rarity, IUCN conservation status and species richness by region were combined. Four main regions have been identified through the distribution analysis of vulnerable reptile species:

1. A large region in the north of the country extends over the large coastal mountain ranges and contains the Edough Massif, the Babors and Djurdjura. These regions include threatened and vulnerable species.

- *Mauremysleprosa* (Vulnerable);
- *Testudograeca* (Vulnerable);
- *Emysorbicularis* (Near Threatened);
- *Viperamonticola* (Vulnerable)

2. The Mountains of Aures represent the region in Algeria with the highest reptile diversity. It includes threatened, rare and endemic species:

- *Mauremysleprosa* (Vulnerable);
- *Testudograeca* (Vulnerable);
- *Acanthodactylusbedriagai* (endemic);
- *Viperamonticola* (rare, Vulnerable);
- *Tropiocoloteschirioi* (Data Deficient);
- *Ophisopselegans* (Data Deficient).

3. Northwest Region (Oranie)

- *Mauremysleprosa* (Vulnerable);
- *Testudograecagraeca* (Vulnerable);
- *Chalcidesminutus* (Vulnerable);
- *Chalcidesparallelus* (Endangered);
- *Chalcidesmauritanicus* (Endangered);
- *Scelarcisperspicillata* (endemic);
- *Hyalosauruskoellikeri* (endemic);
- *Eumecesalgeriensis* (endemic).

4. A fourth zone in the middle of the Sahara represented by the Massifs of Tassili n’Ajjer and Ahaggar harbouring a particular herpetofauna with rare and threatened species:

- *Uromastyxalfredschmidti* (Near Threatened);
- *Tropiocolotestassiliensis* (Data Deficient);
- *Trapelusschmitzi* (Data Deficient);
- *Uromastyxgeyri* (Near Threatened);
- *Philochortuszolii* (Endangered);
- *Psammophisaegyptius* (Data Deficient).

Fig. 2

## Discussion

The distribution of reptiles is related to several factors, mainly habitat diversity and also climatic factors. With an average of 45 species, the Aurès Mountains are the most diversified region of northern Algeria. The topography and the mountainous character of the region, as well as the diversity of its plant cover and the Mediterranean and Saharan climatic influences, have allowed a diversity of reptilian fauna to exist here. Indeed, this area is a real junction where desert and Mediterranean species co-exist.

### Sea turtles

The presence of sea turtles along the Algerian coast is regularly reported, with many beachings, of which 70% are the loggerhead turtle, *Carettacaretta* (Linnaeus, 1758) and 30% of the leatherback Sea turtle, *Dermochelyscoriacea* (Vandelli, 1761) (Bennounas and Bennounas 2020, Belmahi et al. 2020). The green Sea turtle *Cheloniamydas* (Linnaeus, 1758) is the rarest species, having been recorded only once since 2003 (Bennounas and Tifoura 2020). The first study of sea turtle nesting on the Algerian coast dates back to 1998 (Laurent 1990), when surveys were unsuccessful and no nests were found. Since then, nesting attempts have been reported on the east coast of Algeria, most recently in 2017, when a loggerhead nest was discovered in Collo (Skikda) (Benabdi and Belmahi 2020). In the summer of 2023, loggerhead nesting was confirmed on the Algerian coast, with hatchlings observed on the "Aftissen" beach of Béni Fergane in the wilaya of Jijel, still in eastern Algeria (Ahmim et al. 2024). Newly-hatched loggerheads were also reported on a beach in Algiers. This confirms the conclusion of Carreras et al. (2018) that these nests represent a new colonisation of the Western Basin by this species of sea turtle, mainly as a result of climate change impacts on migration and reproduction. There may be other nesting sites along Algeria's 1,000 km coastline that have not been identified because there is no dedicated sea turtle monitoring instance.

### Testudograeca Linnaeus, 1758

The Spur-thighed tortoises (*Testudograeca*) represent the most widely distributed species of tortoise in the Western Palaearctic (Buskirk et al. 2001, Iverson 1992). It is the only terrestrial species that exists in Algeria; it has a wide distribution from the north to the limit of the Saharan Atlas. Phylogeography of North African populations of *T.graeca* has received important interest (Graciá et al. 2017, Fritz et al. 2009, Salinas et al. 2009, Fritz et al. 2007, van der Kuyl et al. 2005, Pieh 2000). Actualy, *T.graeca* comprises five subspecies: *Testudograecagraeca* (Linnaeus, 1758), older synonym of *T.g.soussensis* Pieh, 2000; *Testudograecacyrenaica* Pieh & Perälä, 2002; *Testudograecamarokkensis* Pieh & Perälä, 2004; *Testudograecanabeulensis* Highfield, 1990 and *Testudograecawhitei* (Bennett in White (1836)). It was previously thought that the nominotypical form *T.g.graeca* occurred in the Iberian Peninsula (Alvarez et al. 2000), but Schweiger and Gemel (2020) corrected the confusing history regarding the type locality of *T.g.graeca*, documenting it as Agadir in south-western Morocco, thereby rendering *T.g.whitei* the apparently most correct name for the subspecies on the Iberian Peninsula and north-eastern Morocco and western Algeria (Uetz 2021). In Algeria, two subspecies are identified: *Testudograecanabeulensis* occurs in eastern Algeria, inhabiting mainly humid to semi-arid Mediterranean climates and *T.g.whitei* occurs mainly in Algeria (Graciá et al. 2017) in mixed oromediterranean forests of *Cedrusatlantica* (Endl.) Manetti ex Carrière, 1855 and *Quercusilex* Linnaeus, 1753 (Escoriza et al. 2022). This species is considered endangered across its entire range. In Algeria, the capture and trade of this species has been illegal since 1983 (Décret No. 83-509). This species is threatened by the destruction of its habitats and by the trade, particularly in hatchling and juvenile turtles which are commonly traded in markets as household pets (Rouag 2016, Atoussi et al. 2022).

### Genus Acanthodacylus

*Acanthodactylus* Fitzinger, 1834 constitutes the most species-rich genus in the family Lacertidae, with over 40 recognised species inhabiting a wide variety of dry habitats. The genus has seldom undergone taxonomic revisions and, although there are a number of described species and species-groups, their boundaries, as well as their interspecific relationships, remain largely unresolved (Tamar et al. 2016). The situation in North Africa is complex and the relationships with other species unclear (Harris et al. 2004, Fonseca et al. 2009, Psonis et al. 2016).

In North Africa, *Acanthodactylus* species are divided within three clades: the *erythrurus* and *pardalis* groups occupying the Sub-Saharan Region and the coastal areas of North Africa; the *scutellatus* group, occurring mainly in the sandy areas of North Africa (Tamar et al. 2016).

***Acanthodactyluserythrurus* species complex**

*Acanthodactyluserythrurus* (Schinz, 1833), *A.lineomaculatus* Duméril & Bibron, 1839 and *A.blanci* Doumergue, 1901 are part of a group called «*A.erythrurus* species complex» whose taxonomy is complex and unstable because of their wide distribution and also their great genetic variation (Fonseca et al. 2009).

The Spiny-footed Lizard, *Acanthodactyluserythrurus* is widespread in the Iberian Peninsula and the Maghreb, from Morocco to Tunisia (Fonseca et al. 2009). Numerous attempts have been made to subdivide it into subspecies on the basis of phylogenetic studies (Fonseca et al. 2009, Harris et al. 2004). Miralles et al. (2020) highlighted the existence of five clades in the Maghreb where the divergence between them is broadly similar, supporting the existence of at least five species in the *Acanthodactyluserythrurus* complex: an Ibero-Moroccan clade, a Central Algerian clade (found on the Mediterranean coast, formed by two inland populations situated north and south of the High Plateaux, near Theniet-El-Had and around Djelfa), an Algero-Tunisian clade (Tunisia and coastal populations of eastern Algeria extending from Zemmouri to Sidi Abdelazziz, including populations described under the name *Acanthodactylusblanci*) and two clades from the Eastern and Western High Atlas newly described as *Acanthodactyluslacrymae* Miralles et al., 2020 and *Acanthodactylusmontanus* Miralles et al., 2020 (Miralles et al. 2020).

***Acanthodactyluspardalis* group**

In northern Africa, with the exception of *Acanthodactylusspinicauda* Doumergue, 1901, the systematics of this group is not clear and there is profound disagreement amongst authors (Fonseca et al. 2008). According to Salvador (1982), this group includes five species: *A.pardalis* (Lichtenstein, 1823), *A.busacki* Salvador, 1982, *A.maculatus* (Gray, 1838), *A.bedriagai* Lataste, 1881 and *A.spinicauda* Doumergue, 1901. *A.maculatus* is covering the Algerian High Plateaux; *A.bedriagai* is restricted to the Oriental Plateaux of Algeria and *A.spinicauda* known only from a place called “Berr’mad” situated 50 km south of El Abiod Sidi Cheikh. However, the systematics of *Acanthodactylus* of the *pardalis* group in the Maghreb needs to be revised as it presents high levels of intraspecific variability and clear evidence of phylogenetic complexity such as *A.maculatus* and *A.bedriagai* populations of the eastern Morocco and Algeria. Tamar et al. (2016) confirmed that *A.maculatus* complex is not a monophyletic taxon, but corresponds to two species (*A.bedriagai* and *A.maculatus*) forming a paraphyletic group.

***Acanthodactylusscutellatus* group**

The *Acanthodactylusscutellatus* species group comprises seven recognised species (*A.aegyptius* Baha El Dine, 2007, *A.aureus* Gûnther, 1903, *A.dumerilii* Miline-Edwards, 1829, *A.longipes* Boulenger, 1918, *A.scutellatus* Audouin, 1827, *A.senegalensis* Chabanaud, 1918 and *A.taghitensis* Geniez and Foucart, 1995) which are abundant and conspicuous across xeric environments of North Africa and the Middle East (Liz et al. 2022). This group is represented in Algeria by *Acanthodactylustaghitensis*, *Acanthodactyluslongipes*, *Acanthodactylusdumerilii* and the nominate species *A.scutellatus* which is represented by the monophyletic lineage corresponding to the subspecies *A.s.audouini* Boulenger, 1918 (Tamar et al. 2016). Most species are linked to sandy habitats, but their ecology varies from the soft-sand specialist *A.longipes* to the more generalist *A.scutellatus*. The only exception is *A.taghitensis*, which only occurs in gravel plains (Liz et al. 2022).

***Acanthodactylusboskianus* group**

The boskianus group is represented by two paraphyletic species, *Acanthodactylusboskianus* in North Africa and *Acanthodactylusschreiberi* Boulenger, 1878 in the Middle East (Tamar et al. 2014). *Acanthodactylusboskianus* is the most widely distributed species of the genus, ranging from Morocco through North Africa to Iran (Uetz 2021). Five morphological subspecies are currently recognised in Bosk's fringe-fingered lizard (Uetz 2021). *A.boskianusasper* (Audouin, 1827) is the only representative of this group in Algeria (Tamar et al. 2016). It has a wide distribution in sandy habitats.

### Genus Mesalina

*Mesalina* species are small, fast, ground-dwelling, diurnal lizards, well-adapted to desert and xeric shrublands. All *Mesalina* taxa can be divided according to their phylogenetic relationships into the following seven assemblages: *M.watsonana* (Stoliczka, 1872), *M.martini* (Boulenger, 1897), *M.rubropunctata* (Lichtenstein, 1823), the *M.adramitana* group, the *M.brevirostris* group, the *M.guttulata* group and *M.olivieri* (Simó-Riudalbas et al. 2019, Pizzigalli et al. 2021). In Algeria, we record *M.guttulata* (Lichtenstein, 1823) and *M.olivieri* (Audouin, 1829) which are a species complex including a main clade in the Middle East and another in North Africa (Nouira et al. 2022). *M.rubropunctata* (Lichtenstein, 1823) and *M.pasteuri* (Bons, 1960) are monophyletics and occupy most of the Algerian Sahara. *M.rubropunctata* affects arid areas with stony or rocky soil, rarely sandy, whereas *Mesalinapasteuri* occupies the driest regions of the Sahara and is found in the dunes of ergs as well as in plains with more compact sandy soils (Trape et al. 2012).

### Genus Tarentola

Algeria harbours five different species of *Tarentola*, namely: *T.mauritanica* (Linnaeus, 1758), *T.deserti* Boulenger, 1891, *T.neglecta* Strauch, 1887, *T.annularis* (Geoffroy Saint-Hilaire, 1827) *and T.hoggarensis* Werner, 1937, recently elevated to the rank of species based on molecular and morphological data (Trape et al. 2012). *Tarentolamauritanica* is characterised by an extremely high mitochondrial genetic variation in North Africa, which led to the hypothesis that this taxon could be, in fact, a species complex (Harris et al. 2004). In Algeria, its distribution is wide in the northern part up to the Saharan Atlas where it occupies rocky habitats and tree formations; discreetly anthropophilic, it frequents habitations. The Desert Wall Gecko *Tarentoladeserti* is a Saharan species endemic to the Maghreb (Joger et al. 2006) not usually present in areas with rainfall below 100 mm annually (Schleich et al. 1996). In Algeria, it is widely distributed in the northern Saharan oases, in the Saharan Atlas and on the High Plateaus. It inhabits rocky deserts (regs), sandy deserts (ergs), dry wadis, palm oases and ruins, where they usually hide in cracks and crevices. Two subspecies have been described from *T.neglecta* Strauch, 1887: *Tarentolaneglecta neglecta S*trauch, 1895, in the south of the Sahara-Atlas from Algeria, Tunisia to west Libya and *Tarentolaneglectageyri* Joger, 1984 which occurs in south Algeria in central Sahara, from the south-western edge of the great eastern Erg to the foothills of the Ahaggar Mountains; the exact delimitation of the distribution area is still unclear (Joger 1984).

### Genus Cyrtopodion

This new genus for Algerian herpetofauna, considered invasive, is represented by *Cyrtopodionscabrum* (Heyden, 1827). This species is widely distributed in Afghanistan, Egypt, Ethiopia, India, Iraq, Palestine, Jordan, Kuwait, Oman, Pakistan, Qatar, Saudi Arabia, Sudan, Syrian Arab Republic, Turkey, United Arab Emirates and Yemen) (Khan 2005, Khan 2008, Cogălniceanu et al. 2014). Moreover, this species is also introduced outside of its native geographical range in Iran Islamic Republic (Rastegar-Pouyani et al. 2010) and in Texas and Nevada in the United States (Bartlett and Bartlett 1999, Stocking and Jones 2017). The first documented record of *C.scabrum* in Algeria was on 27 June 2009 in the north-eastern Sahara from five different locations at El Oued (Mouane 2020) and then also in El Menea (Sadine et al. 2021). A new locality was recorded from Ouargla Province, south-east Algeria (Mouane 2022). The causes of *C.scabrum's* invasion are most likely due to an accidental introduction linked to the importation of various agricultural products which constitute the main commerce in this region (Mouane 2020). *C.scabrum* is currently more abundant in sites where agricultural activities are important (expanding irrigated lands), specifically the urban areas near date palm plantations (Mouane 2020).

### Genus Tropiocolotes

A total of 12 species are recognised within the genus *Tropiocolotes*, covering a distribution range from Atlantic Sahara, Maghreb, Levant, Arabian Peninsula and Iran (Machado et al. 2021). *Tropiocolotes* was divided into two highly-divergent groups, one comprising the African clade formed by *Tropiocolotesalgericus* Loveridge, 1947 and *Tropiocolotestripolitanus* Peters, 1880 and the other comprising the Saharo-Arabian clade (*T.nattereri*/ *T.confusus*/ *T.scorteccii*/ *T.naybandensis*/ *T.bisharicus*/ *T.somalicus*/ *T.steudneri*/ *T.nubicus*) (Machado et al. 2018). In Algeria, we found the two groups represented by:

-*Tropiocolotestripolitanus* Peters, 1880, in the Algerian Sahara and reported in Tindouf, Ahaggar, Biskra, Figuig and Kenadsa (Schleich et al. 1996);

-*Tropiocolotesalgericus* Loveridge, 1947, in the westernmost portion of the Saharan Atlas Mountain Range, with a disjunct population in the northern portion of the Tademaît Rocky Plateau (Ribeiro-Júnior et al. 2022). Considered as a subspecies of *T.tripolitanus*, it was elevated to species rank by Baha El Din (2001).

-*Tropiocolotesnubicus* Baha El Din, 1999, in southern Algeria (Tassili n’Ajjer and Ahaggar). Phylogenetic results of Machado et al. (2021) grouped *Tropiocolotessteudneri* from Niger and southern Algeria within *Tropiocolotesnubicus* Baha El Din, 1999, while *T.steudneri* specimens were only found east of the Nile River.

Recently, two new species were reported for Algerian *Tropiocolotes* by Ribeiro-Júnior et al. (2022) on the basis of external morphologyand osteological characters; these are:

-*Tropiocoloteschirioi* Ribeiro-Júnior, Koch, Flecks, Calv & Meiri, 2022; Described on specimens collected by Laurent Chirio. The type-locality is situated in the Aurès Mountains (north-eastern Algeria).

-*Tropiocolotestassiliensis* Ribeiro-Júnior, Koch, Flecks, Calv & Meiri, 2022, in southern Algeria (Tassili n’Ajjer and Ahaggar). The type-locality is situated 3 km east of Tamanrasset on the road to Adriane in the Tassili n’Ajjer mountain (south-eastern Algeria).

### Genus Uromastyx

On molecular biology arguments, Wilms et al. (2009) elevated the subspecies *Uromastyxacanthinurusnigriventris* Rothschild & Hartet, 1912 to the status of a separate species *Uromastyxnigriventris* Rothschild and Hartert, 1912 which is distributed in Morocco and western Algeria (from Mzab to Guir) (Trape et al. 2012). This species is replaced in central and eastern Algeria by the North African spiny-tailed Lizard *Uromastyxacanthinura* Bell, 1825. The species *Uromastyxalfredschmidti* Wilms and Böhme, 2001, is a large lizard occupying the Tassili Massif n' Ajjer in south-eastern Algeria and bordering regions of Libya where it is often associated with areas of large boulders (Trape et al. 2012). The distribution area of Saharan Spiny-tailed Lizard *Uromastyxgeyri* Müller 1922 extends from the mountainous massifs and rocky plateaus of the central Sahara, from the Aïr in Niger to the Adrar des Iforas in Mali towards the North, where it reaches Ahaggar and the Amguid Region of Tassili n’Ajjer in Algeria (Trape et al. 2012). *Uromastyxdisparmaliensis* Joger and Lambert, 1996 also lives in north-western Mali, in the Tilemsi Valley, on the edge of the Adrar des Iforas and in south-western Algeria (Taoudrart en Tanezrouft) (Wilms et al. 2009). *Uromastyxdisparmaliensis* and *U.geyri* are sympatric in the Adrar des Iforas Region (Joger and Lambert 1996). The northernmost locality of *U.d.maliensis* is Gara Djenoum / Monts du Ahaggar (Wilms and Böhme 2001). *Uromastyxdisparflavifasciata* Mertens, 1962 occupies the north of Western Sahara and the Tindouf Region in Algeria near the Moroccan border where it exists in rocky areas and stony plains (Trape et al. 2012).

### Genus Tournevillei / Agama

*Agamabibronii* A. Duméril *in* Duméril & Duméril, 1851 is the valid name for the North African rock agama (Denzer 2021). This species ranges from the Atlantic coastal region of Western Sahara, through most of Morocco to north-western Algeria (Trape et al. 2012). *Agamatassiliensis* (Geniez, 2011) is a newly-described species (Geniez et al. 2011) previously part of the populations of *Agamaimpalearis* Boettger, 1874. The range of this species extends over the large mountain massifs and rocky areas of the central Sahara: Adjar des Ifhoras (Mali), Aïr (Niger) and Tibesti (Chad). In Algeria, it is present in Tassili n'Ajjer and Ahaggar. Another species newly described by Wagner et al. (2011) on the bases of molecular phylogeny and morphology is *Trapelusboehmei* (Wagner 2011) which occurs in Mauritania, Morocco and Algeria where its presence covers the entire Sahara. Previously, it was part of *Trapelusmutabilis* Merrem, 1820 which is found throughout the Saharan desert region, from the Saharan Atlas in the north and to the Saharan-Sudanese borders, from the Atlantic to Egypt. The Sahara Agama *Trapelustournevillei* (Lataste 1880) is located in two distinct parts of central Algeria and is also present in ‘Erg Oriental’ (Touggourt, Ouargla), ‘Erg Occidental’ (El Goléa, Béni Abès) and ‘Erg er Raoui’ (Schleich et al. 1996). *Trapelusschmitzi* Wagner & Böhme, 2007 was recently described on the basis of a single specimen from the Ennedi Mountains (Chad). The second voucher from The Natural History Museum of the City of Geneva (MHNG 901.70) collection was collected by J. Juge in 1952 in Algeria at Tassili n'Ajjer, a 500 km long mountain chain in south-eastern, near the Ahaggar Mountains (Wagner et al. 2008). No data are available on this species in Algeria; it is classified as Data Deficient (DD) on the IUCN Red List.

### Family Scincidae

*Chalcidesminutus* Caputo, 1993, is a little-known species found in northern Morocco. In 2014, Montero-Mendieta et al. discovered a skink belonging to the *Chalcides* genus in Théniet El Had National Park (Algeria). Initially classified as *Chalcidesmertensi* Klausewitz, 1954 due to its morphological similarity and distribution, this skink was surprisingly found to be genetically closely related to specimens of *Chalcidesminutus* (Caputo, 1993). Genetic analysis can help to understand the phylogeny of the *Chalcidesminutus-mertensi* species complex (Montero-Mendieta et al. 2017). *Chalcidesmauritanicus* (Duméril and Bibron 1839) endemic to north-eastern Morocco and north-western Algeria was first described by Doumergue (1901) from the littorial area of north-western Algeria; it is narrowly restricted to coastal districts of the Algiers and Oran Provinces (Pasteur 1981). *Chalcidesparallelus* (Doumergue, 1901) is also endemic to north-eastern Morocco and north-western Algeria occurring mainly along a narrow coastal strip of approximately 250 km between Nador in north-eastern Morocco and Cape Carbón in north-western Algeria. This species has a similar distribution to *Chalcidesmauritanicus*, but is found far from the dune cordon, especially on islands (Beddek 2017). The main distribution of this species is on beaches. Its presence in sandy biotopes indicates the potential dangers of excessive sand extraction from beaches (Beddek 2017). Within the *Sincusscincus* complex, Arnold and Leviton (1977) identified two subspecies in Algeria: *S.s.cucullatus* Werner, 1914, in north-eastern Algeria and *S.s.laterimaculatus* Werner, 1914, in north-western Algeria.

### Genus Macroprotodon

*Macroprotodon* are colubrines that are found in mainly Mediterranean areas of North Africa, the Iberian Peninsula (Iberia) and on some western Mediterranean islands (Doumergue 1901, Busack and McCoy 1990, Wade 2001, Carranza et al. 2004). The taxonomy of this genus in North Africa is still largely unclear and systematic studies integrating genetics and morphology are necessary to clarify the situation (Nouira et al. 2022). In Algeria, Wade (2001) recognises three species: *Macroprotodoncucullatus* (Geoffroy de St Hilaire, 1827) occurs in relatively arid areas and divided in two subspecies: *M.c.cucullatus* and *M.cucullatustextilis* (Duméril and Bibron, 1854), while *M.mauritanicus* Guichenot, 1850 and *M.abubakeri* Wade, 2001, occurs mainly further north and even occupying some islands. *Macroprotodonabubakeri* was recently described by *Wade (2001)* and its status has recently been confirmed from genetic data (Carranza et al. 2004). This species is known from north-western Algeria and also occurs on Habibas Islands. There is very little recent information on this species and it apparently lives in semi-arid and sub-humid Mediterranean habitats. On the other hand, *M.mauritanicus* is distributed according to Busack and McCoy (1990) in north-eastern Algeria. *Macroprotodoncucullatus* requires a taxonomic revision as it appears to be paraphyletic, as indicated by Carranza et al. (2004). *Macroprotodoncucullatustextilis* is the subspecies found in the entire northern part of the Algerian Sahara and isolated population exists also in the Ahaggar in southern Algeria. Geniez (2015), based exclusively on morphology, considers that *M.cucullatustextilis* should be included within *Macroprotodonbrevis* (Günther 1862), which was also reflected in later works (Martínez del Mármol et al. 2019). In the present work, we will also consider this classification. A proper understanding of *M.cucullatus* must await the availability of DNA samples from a much wider range of populations, including ones distant from *M.brevis* and *M.mauritanicus*, such as the isolates in the Ahaggar (Algeria) (Carranza et al. 2004).

### Genus Daboia

Algerian Daboia vipers include two species, *Daboiamauritanica* Gray, 1849 and *Daboiadeserti* (Anderson, 1892) with controversial range delimitations where *Daboiamauritanica* is distributed between the Tellian Atlas and the Saharan Atlas and *Daboiadeserti* occupies a narrow strip of the Saharan Atlas, from western Algeria, towards Tunisia. Based on a molecular study on North African Daboia vipers, Martínez-Freiría et al. (2017) do not support the occurrence of two distinct taxa, revealing that the *deserti* taxon can no longer be admitted as a valid species and all North African species should thus be referred to as *D.mauritanica*. Furthermore, *D.deserti* is identified as an invalid taxon.

### Genus Echis

The genus *Echis* Merrem, 1820 is one of the most complex genera of snakes in Africa, its being found throughout the semi-arid/xeric regions of Western Africa, thence eastwards to southern Asia (Uetz 2021). Recent genetic studies have subdivided the genus *Echis* into four main clades consisting of the *E.ocellatus*, *E.coloratus*, *E.pyramidum* and *E.carinatus* groups (Pook et al. 2009). Within the *E.pyramidum* (Geoffroy Saint-Hilaire, 1827) clade, *E.leucogaster* Roman, 1972 inhabits the western Sahel Region, with possibly isolated populations in the Algerian Ahaggar massif and Tassili n’ajjer (Pook et al. 2009, Geniez 2015). In North Africa, recent genetic analysis has shown that the genetic variability between the *Echisleucogaster* and *Echispyramidum* group is very low and some authors suggest the existence of a single species with several subspecies (Arnold et al. 2009). Due to its low genetic variability, it has been proposed that the species *Echisleucogaster* is considered a subspecies of *Echispyramidum* (Sindaco et al. 2013, Geniez 2015).

### Data deficient and unconfirmed Species

Some of species cited for the Algerian herpetofauna are classified in the Data Defficient category, while others should be noted as "unconfirmed", such as the Puff Adder *Bitisarietans* (Merrem, 1820), which is mentioned by some authors, but for which we have found no bibliographical reference confirming its presence in Algeria. Identification difficulties also occur between taxonomically related species, this being the case of *Stenodactylussthenodactylus* (Lichtenstein, 1823), which is indistinguishable from *Stenodactylusmauritanicus* Guichenot, 1850. Its presence in Algeria has only been reported in the south by Metallinou et al. (2012) in Oued Dider, Aguelmane Assar (Tassili n'Ajjer). Another rare species recorded from southern Algeria is the Egyptian Grass-loving lizard, *Philochortuszolii* (Scortecci, 1934). It was first recorded in May 1974, when T.J. Pappenfuss, R.C. Drewes and E.J. Morris collected two specimens (Scheinberg and Fong 2024), 3 km east of Tamanrasset on the road to Adriane. The specimens are currently at the California Academy of Sciences (CAS). This is a very rare species, which occur in small sub-populations. New observations were made recently in the Municipality of Tagmart-East, in the wilaya of Tamanrasset on 23 July 2023 by Haddad et al. (2024), confirming the presence of this species in Algeria. The Egyptian Catsnake *Telescopusobtusus* (Reuss, 1834) is a poorly-known species, little data exist on its distribution in Algeria; the only stations cited in the literature are located in Mertoutek (Ahaggar) and In-Sebuk Oua Mellen (Immidir), those populations in the mountains of southern Algeria being presumably isolated (Crochet et al. 2008). Another viper reported in Algeria is suspected of being part of the genus *Daboia*; it is, in fact, The Levant Viper *Macroviperalebetinus* (Linnaeus, 1758). Specimens from northern Algeria and Tunisia, preserved for a long time in museums, have been attached to the subspecies *Macroviperalebetinustransmediterranea* (Nilson & Andrén 1988). One of the few specific localities is Djebel Murdjajo near Oran in western Algeria. The validity of the taxa *M.l.transmediterranea* as full species is currently uncertain due to the scarcity of records along all of its supposed distribution (Jiménez Robles and del Mármol Marín 2012). It is not impossible that the presence of *M.lebetinus* in North Africa results from introductions from Asia Minor during Antiquity (Martínez-Freiria pers. comm. in Nouira et al. (2022)). A new species has just been identified in the Algerian herpetofauna: The Saharan Sand Snake, *Psammophisaegyptius* Marx, 1958. This species, already described in south-eastern Algeria by Schleich et al. (1996) and in the Ahaggar by Trape and Mané (2006), was recently reported by Boulaouad A. on 21 December 2021 at In Guezzam (Tamanrasset), near the border with Niger (https://www.inaturalist.org/observations/185341814).

### Conservation strategy for Reptiles

Many reptiles are facing threats due to habitat degradation, but the current conservation measures are insufficient to address the critical concerns for their survival. The studies and reports on biodiversity in Algeria are focused on the fauna as a whole, without carrying out specific studies on the state of conservation of the herpetological communities and their habitats. Additionally, Algerian legislation does not offer total protection to those species and their habitat. Incentives and legislative measures must be established. Thus, the protection of Algerian reptiles requires a better knowledge of their ecology and distribution. The deficiencies of knowledge of their taxonomy, biology, dynamics and rate of evolution are significant. The status of several species remains to be defined. This lack of data limits the conservation of these species and makes their management quite difficult. Therefore, it is fundamental to establish a national strategy for better knowledge and thus better conservation of this fauna.

## Figures and Tables

**Figure 2a.**
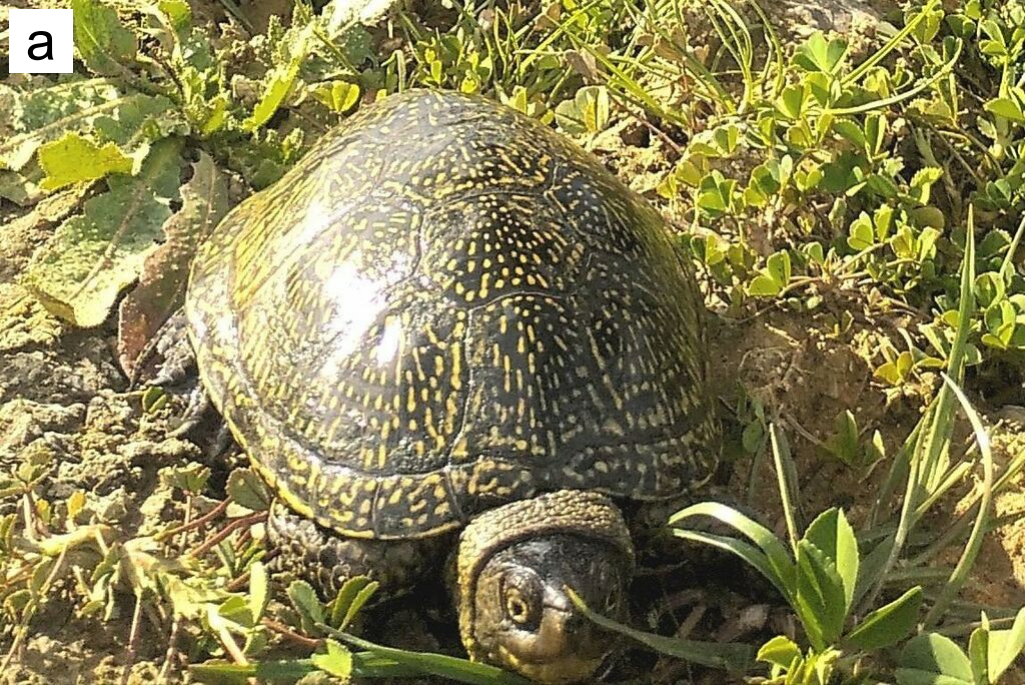
*Emysorbicularis* (Annaba, July 2023) (Photo by R. Rouag);

**Figure 2b.**
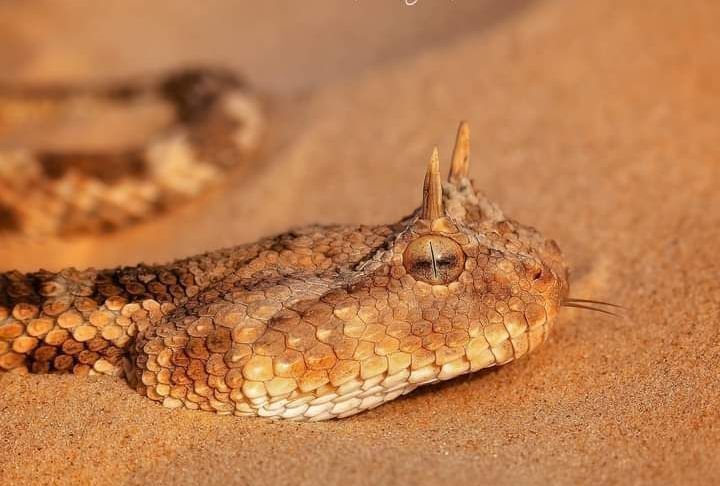
*Cerastescerastes* (Oued Souf, September 2023) (Photo by S. Sidali);

**Figure 2c.**
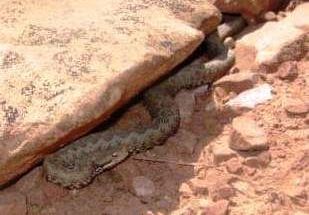
*Viperamonticola* (Djurdjura National Park, June 2012) (Photo by R. Rouag);

**Figure 2d.**
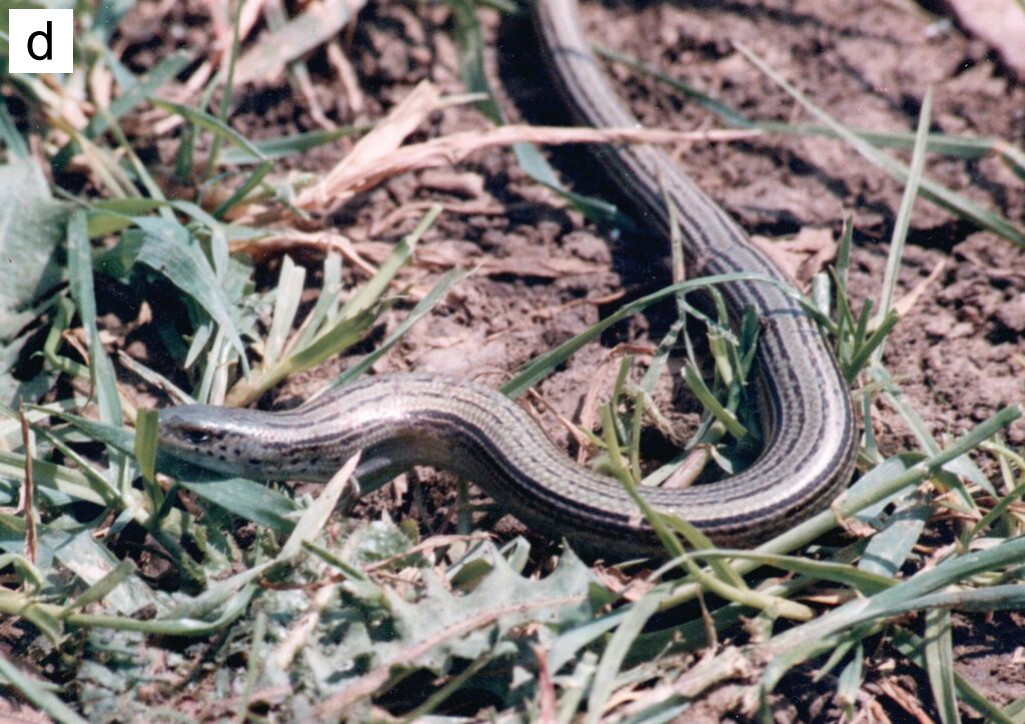
*Chalcidesmertensi* (El Kala National Park, July 2000) (Photo by S. Benyacoub).

**Table 1.** Diversity in reptile orders and families within Algeria.

**Orders**	**Suborders**	**Families**	**Species**
Testudines	Cryptodira	5	6
Squamata	Sauria	11	71
	Serpentes	8	27
**Total**		**24**	**104**

**Table 2.** Summary of the global Red List status for Reptiles of Algeria.

**Least Concern (LC)**	**Near Threatened (NT)**	**Vulnerable (VU)**	**Endangered (EN)**	**Critically Endangered (CR)**	**Data Deficient (DD)**	**Not Evaluated (NE)**
73	06	05	04	01	15	0

**Table 3.** Status of sensitive species.

**Species**	**Endemic**	**Population trend**	**Geographic range**
*Acanthodactylusbedriagai*	Algeria	Unknown	Northeast (Aurès)
*Acanthodactylussavigny*	Algeria	Unknown	Northeast (Coastal regions)
*Tropiocoloteschirioi*	Algeria	Unknown	Northeast (Aurès)
*Tropiocolotestassiliensis*	Algeria	Unknown	Tassili n’Ajjer
*Chalcidesmertensi*	Algeria-Tunisia	Unknown	Northern
*Timonpater*	Algeria-Tunisia	Decreasing	Northeast
*Tarentolaneglecta*	Algeria-Tunisia	Stable	Saharan
*Timontangitanus*	Algeria - Morocco	Decreasing	Northwest
*Scelarcisperspicillata*	Algeria - Morocco	Stable	Northwest
*Hyalosauruskoellikeri*	Algeria – Morocco	Unknown	Northwest
*Trapelustournevillei*	Algeria - Morocco	Stable	Saharan
*Chalcidesminutus*	Algeria - Morocco	Decreasing	Tlemcen Mountains
*Chalcidesparallelus*	Algeria - Morocco	Decreasing	Northwest
*Chalcidesmauritanicus*	Algeria - Morocco	Decreasing	Oran-Algiers (Coastal regions)
*Eumecesalgeriensis*	Algeria - Morocco	Stable	Northwest
*Viperamonticola*	Algeria - Morocco	Decreasing	Tellian Atlas
*Saurodactylusmauritanicus*	Algeria - Morocco	Decreasing	Northwest
*Uromastyxalfredschmidti*	Algeria - Libya	Stable	Southern (Tassili n’Ajjer)
